# Preliminary lipidomics and transcriptomics reveals stage-specific dynamic metabolic patterns from menopause transition to postmenopause

**DOI:** 10.3389/fendo.2026.1726161

**Published:** 2026-03-03

**Authors:** Qun Zhan, Miao-Miao Li, Xia Chen, Run-Ze Feng, Qing-Ling Ren, Qiu Du

**Affiliations:** 1 Department of Gynecology, Nanjing Hospital of Chinese Medicine affiliated to Nanjing University of Chinese Medicine, Nanjing, Jiangsu, China; 2 School of Graduate, Nanjing University of Chinese Medicine, Nanjing, China; 3 Department of Gynecology, Jiangsu Province Hospital of Chinese Medicine, Affiliated Hospital of Nanjing University of Chinese Medicine, Nanjing, Jiangsu, China; 4 Department of Pharmacy, Nanjing Hospital of Chinese Medicine, Nanjing, Jiangsu, China

**Keywords:** leukocyte transcriptomics, lipid molecules, lipidomics, marker genes, menopausal stages

## Abstract

**Objective:**

This study aimed to systematically analyze the clinical indicators, lipid metabolism, and leukocyte transcriptomic profiles of premenopausal, menopause transition, and postmenopausal women. It sought to explore stage-specific characteristics of lipid metabolism and gene expression and their potential associations with key clinical indicators.

**Methods:**

Clinical indicators were collected, and untargeted lipidomics and leukocyte transcriptomic sequencing were performed. Multivariate statistical analyses were used to assess intergroup differences. Significant patterns in lipid and gene expression changes were identified through trend clustering, volcano plots, and Venn diagram analyses. GO and KEGG enrichment analyses were conducted to investigate the biological functions and pathway enrichment of differentially expressed genes.

**Results:**

With advancing menopausal stages, E2 levels were lower, while FSH and LH levels were higher. BMI was higher during the menopause transition and subsequently declined, whereas CHOL and LDLC levels were higher in both the menopause transition and postmenopause stages.Specific lipid classes showed partial return to baseline or further regulation in postmenopause. Transcriptomic analysis identified stage-specific differentially expressed genes enriched in inflammation- and lipid metabolism-related pathways. E2 was positively correlated with lipid molecules and marker genes, but negatively correlated with CHOL.

**Conclusion:**

This preliminary study indicates that menopausal stages are associated with significant stage-specific differences in lipid metabolism and gene expression, with the menopause transition identified as a critical phase of lipid metabolic reorganization. Certain lipids and genes may serve as potential biomarkers for menopause-associated metabolic differences. These findings provide important insights for early warning and intervention strategies targeting menopause-related health issues.

## 1 Introduction

The perimenopausal stage marks a transitional period characterized by declining ovarian function and dysregulation of the hypothalamic-pituitary-ovarian axis, leading to hormonal fluctuations and the onset of menopausal syndrome (MPS) (1). Symptoms such as hot flashes, night sweats, insomnia, and mood disturbances significantly impact quality of life (2). Menopausal syndrome refers to a collection of vasomotor, somatic, and psychological symptoms resulting from hormonal changes during the menopause transition, and varies widely in severity and duration among individuals. Furthermore, MPS has been associated with an higher risk of chronic conditions, including cardiovascular disease (3), diabetes (4), osteoporosis (5), and breast cancer (6), highlighting the need for effective strategies to manage menopausal symptoms and mitigate associated health risks (7).

The term “perimenopause” has replaced “menopause” to more accurately describe the physiological transition surrounding the cessation of menstruation. Perimenopause is divided into three distinct stages: premenopause (regular menstrual cycles within the past 12 months), the menopause transition (≤12 cycles in the past 12 months), and early postmenopause (no menstruation for 12 consecutive months) (8, 9). The World Health Organization (WHO) defines this period as one of declining ovarian follicular function, marked by significant reductions in estrogen and progesterone levels and the emergence of related symptoms (10). Perimenopause typically occurs between the ages of 40 and 60, with most women transitioning around the age of 50 (11).

Altered lipid metabolism has been identified as a critical factor contributing to the pathogenesis of perimenopause-associated conditions (12). While previous studies have explored metabolic changes before and after menopause, many have grouped perimenopausal and postmenopausal stages together, overlooking the unique metabolic characteristics of each phase (13). Advances in multi-omics technologies have enabled the integration of lipidomics and transcriptomics, providing novel tools for investigating the molecular mechanisms underlying the transition from perimenopause to postmenopause (14). Despite this progress, few studies have combined lipidomics and transcriptomics to comprehensively characterize metabolic reprogramming and its potential molecular drivers during the perimenopause transition.

In this study, we systematically analyzed lipidomic and transcriptomic profiles across three stages: premenopause, menopause transition, and postmenopause. Lipidomics was utilized to identify key lipid molecules, while transcriptomics revealed functional changes in lipid metabolism-associated genes. Additionally, we examined the relationships between lipid molecules, hormone levels, and gene expression. Our findings provide new insights into the molecular mechanisms of metabolic remodeling during perimenopause and offer a foundation for personalized therapeutic strategies targeting perimenopause-associated conditions.

## 2 Materials and methods

### 2.1 Study population

This study included women in premenopause, menopause transition, and postmenopause stages, selected based on strict inclusion and exclusion criteria to ensure data representativeness and reliability. Participants were recruited between July 2023 and July 2024 from Nanjing Hospital of Chinese Medicine (The Nanjing Ethics Committee of Traditional Chinese Medicine approved the study with the ethical approval number: KY2023070), located in Nanjing China, during routine health screenings and outpatient visits. Recruitment was conducted through voluntary enrollment after informed consent.

Classification into the three menopausal stages was based on the STRAW + 10 criteria, using menstrual history as the primary criterion and hormonal levels as supportive data. The groups were defined as follows: Premenopause (Pre): women aged 25–45 years with regular menstrual cycles (≥12 cycles/year) in the past 12 months. For premenopausal women, menstrual cycling was prospectively monitored, and blood sampling was standardized to the early follicular phase, prior to the predicted ovulatory window, to minimize hormonal variability. Cycle phase was supported by menstrual history and concurrent serum hormone profiles. Menopause transition (Trans): women aged 45–55 years with irregular cycles (≤12 in the past 12 months). Postmenopause (Post): women aged 50–65 years with no menstruation for ≥12 consecutive months. These ranges reflect the biological staging boundaries recommended by STRAW + 10, rather than artificial age matching. Although menopausal stage and chronological age are biologically correlated, participant stratification was primarily based on STRAW + 10 hormonal and menstrual criteria rather than age matching, in order to emphasize endocrine status. Nevertheless, residual age-related confounding cannot be completely excluded.

Inclusion criteria further required confirmation of serum hormone levels, including estradiol (E2), follicle-stimulating hormone (FSH), and luteinizing hormone (LH) to support stage classification. Exclusion criteria included: (1) severe endocrine or metabolic diseases (e.g., diabetes, thyroid disorders); (2) recent use of hormone therapy (within the past 6 months); (3) medications affecting metabolism (e.g., statins, antibiotics). A total of 90 women (n = 30 per group) were enrolled. Sample size estimation was based on pilot untargeted lipidomics data (n = 12 per group), in which the standardized effect sizes (Cohen’s d) of major lipid subclasses (TG, PC, PE, and Hex1Cer) ranged from 0.65 to 0.82. Power analysis was performed using G*Power 3.1 (one-way ANOVA, fixed effects, α = 0.05), indicating that a minimum of 27 participants per group would achieve >80% statistical power. Therefore, 30 participants per group were recruited to accommodate potential variability and ensure analytical robustness.

### 2.2 Data collection

#### 2.2.1 Clinical and hormonal data collection

Baseline clinical data, including age, height, weight, and body mass index (BMI), were collected during physical examination. Fasting venous blood samples were obtained between 8:30 and 10:00 a.m. to minimize circadian and dietary effects on metabolic indicators. All samples were analyzed in the central clinical laboratory of Nanjing Hospital of Chinese Medicine using standardized protocols. Serum estradiol (E2), follicle-stimulating hormone (FSH), and luteinizing hormone (LH) levels were determined using standardized chemiluminescence immunoassay methods in the hospital’s clinical laboratory.

#### 2.2.2 Other blood measurements

Fasting blood glucose (FBG), total cholesterol (CHOL), triglycerides (TG), high-density lipoprotein cholesterol (HDLC), low-density lipoprotein cholesterol (LDLC), and white blood cell count (WBC) were analyzed as part of routine clinical blood panels using standardized laboratory protocols. All assays were performed by trained personnel within the hospital’s certified laboratory following institutional procedures.

#### 2.2.3 Sample collection and processing

For transcriptomic analysis, we selected a subset of 12 representative samples per group (total n = 36) based on hormone level distribution, demographic consistency, and clinical similarity. Prior saturation testing using DESeq2 on pilot samples demonstrated that this number was sufficient to capture a stable number of differentially expressed genes at FDR < 0.05. Venous blood samples were collected under sterile conditions, with serum separated for lipidomic analysis and leukocytes isolated for transcriptomic analysis. Serum samples were stored at −80 °C, and leukocyte samples were preserved in RNA stabilization solution before storage at −80 °C for subsequent analyses.

### 2.3 Lipidomics analysis

#### 2.3.1 Sample preparation

A total of 90 serum samples (30 from each group: Premenopause/Pre, Transition phase/Trans, and Post-menopause/Post) underwent protein precipitation and ultrasonication to ensure complete metabolite release. The processed samples were vacuum-centrifuged, dried, and reconstituted for analysis.

#### 2.3.2 Lipidomics platform

Untargeted lipidomics was performed using an ultra-performance liquid chromatography (UPLC)-Orbitrap mass spectrometry platform. Lipids were identified and quantified using LipidSearch software (Thermo Scientific™) (Thermo Fisher Scientific, Waltham, MA, USA). Instruments included a Q-Exactive Plus mass spectrometer (Thermo Scientific™) (Thermo Fisher Scientific, Waltham, MA, USA), Nexera LC-30A UPLC (SHIMADZU) (Shimadzu Corporation, Kyoto, Japan), and a Waters ACQUITY UPLC CSH C18 column (1.7 µm, 2.1 mm × 100 mm) (Waters Corporation, Milford, MA, USA). Samples were processed with methanol-acetonitrile solvents and analyzed in randomized order to mitigate signal variation.

#### 2.3.3 Chromatographic and mass spectrometric conditions

Chromatographic separation was achieved on a C18 column at 45 °C with a flow rate of 300 µL/min. Gradient elution was performed with mobile phases A (acetonitrile-water with 0.1% formic acid and 0.1 mM ammonium formate) and B (acetonitrile-isopropanol with 0.1% formic acid and 0.1 mM ammonium formate). Mass spectrometry was conducted in both positive and negative ion modes, with resolutions of 70,000 (MS1) and 17,500 (MS2).

#### 2.3.4 Data processing

LipidSearch was used for peak extraction, lipid identification, alignment, and quantification. Analyses included: (1) lipid classification and composition analysis visualized via bar and pie charts, (2) clustering of lipid expression patterns using Mfuzz software, (3) differential lipid analysis using volcano plots and heatmaps, (4) Venn diagram comparisons of group-specific and shared lipids, (5) multivariate analyses (PCA, PLS-DA, and OPLS-DA) to visualize group separation.

#### 2.3.5 Quality control and batch effect assessment

A pooled QC sample, prepared by combining equal aliquots of all serum samples, was analyzed once every 10 injections to monitor platform stability. Analytical performance was evaluated using the relative standard deviation (RSD) of internal standards and representative lipid species, with >85% of features showing RSD <15%. QC samples clustered tightly in PCA, indicating high reproducibility. Raw intensities were median-normalized, and no obvious batch-related drift was observed.

### 2.4 Transcriptomics analysis

#### 2.4.1 RNA extraction and quality control

Leukocyte RNA was extracted from 12 randomly selected participants in each group using TRIzol commercial kits (Invitrogen, Carlsbad, CA, USA). RNA quality was assessed via NanoDrop (Thermo Fisher Scientific, Waltham, MA, USA) and Qubit (Thermo Fisher Scientific, Waltham, MA, USA) to ensure an A260/A280 ratio of 1.8–2.0.

#### 2.4.2 RNA sequencing

RNA sequencing was performed on the Illumina platform (Illumina Inc., San Diego, CA, USA). Enriched mRNA was used to construct cDNA libraries, followed by paired-end sequencing with a depth of 20–30 million reads per sample. High-quality reads were mapped to the human reference genome (GRCh38) using HISAT2 (Johns Hopkins University, Baltimore, MD, USA) or STAR (Cold Spring Harbor Laboratory, Cold Spring Harbor, NY, USA).

#### 2.4.3 Data processing

Low-quality reads and adapters were removed with FASTQC. Differential gene expression analysis was conducted with DESeq2 or edgeR, using thresholds of p < 0.05 and |log2FC| > 1. GO and KEGG enrichment analyses were performed to identify biological functions. STRING database and Cytoscape software were used to visualize protein-protein interaction networks, highlighting key genes.

### 2.5 Correlation analysis

Twelve factors were selected for correlation analysis based on upstream results from the present dataset to provide an interpretable, stage-linked integrative view. Specifically, we included four clinical indicators (E2, FSH, LH, CHOL) because they represent the endocrine staging anchors and key cardiometabolic readouts analyzed across groups. Four lipid molecules (TG(24:2_11:4_18:2)+NH4, Hex1Cer(t41:1)-H, PC(18:2e_22:5)+H, and PE(18:2e_22:4)+H) were chosen as representative species from the intersection set of shared differentially expressed lipids and were further used in the species-level comparisons and E2–lipid association analysis. Four genes (CMPK2, MX1, RSAD2, IFI6) were selected because they were identified as shared hub genes in the PPI network analyses and exhibited consistent stage-associated expression changes. Pearson or Spearman correlations were applied based on data distribution, with significance set at p < 0.05. Results were visualized using heatmaps and scatter plots in GraphPad 9.0 and R software.

### 2.6 Statistical analysis

All statistical analyses were conducted using R software (version 4.3.0), GraphPad Prism 9.0, and related packages. Continuous variables are presented as mean ± standard deviation (SD). Group differences in clinical indicators (e.g., BMI, hormone levels, blood lipids) were assessed using one-way analysis of variance (ANOVA) followed by Tukey’s honest significant difference (HSD) *post-hoc* test. Statistical significance was set at p < 0.05. For lipidomics data, principal component analysis (PCA), partial least squares discriminant analysis (PLS-DA), and orthogonal PLS-DA (OPLS-DA) were used for dimensionality reduction and group separation. Differential lipid species were identified using a combined threshold of p < 0.01 and false discovery rate (FDR) < 0.05 (Benjamini-Hochberg correction). Volcano plots, heatmaps, and Venn diagrams were employed to visualize lipid species differences and overlaps. Transcriptomic data were processed using the DESeq2 and edgeR packages. Differentially expressed genes (DEGs) were defined as those with p < 0.01 and adjusted FDR (Benjamini-Hochberg) < 0.05. Functional enrichment (GO and KEGG) and gene set enrichment analyses (GSEA) were conducted using clusterProfiler and visualized in R. For correlation analyses among selected hormones, lipids, and genes, Pearson or Spearman coefficients were calculated based on data distribution. FDR correction was applied to all correlation results to control for multiple testing. No AI tools were used in the writing of this manuscript, nor in the creation of figures or in the analysis of data. All content was generated and verified solely by the authors. To reduce method-dependent analytical bias, both unsupervised and supervised multivariate models, together with univariate and pathway-level analyses, were applied in parallel, and only concordant biological trends observed across these complementary statistical layers were interpreted.

## 3 Results

### 3.1 Changes in clinical indicators and hormone levels across menopausal stages

A total of 90 women were enrolled in the study and categorized into premenopause, menopause transition, and postmenopause groups (30 participants each) based on menstrual history and hormone levels. Group differences in age, hormone levels, and metabolic parameters are summarized in **Table 1**. E2 levels were lower, while FSH and LH levels were higher across menopausal stages (p < 0.05). Weight and BMI were higher in the menopause transition group compared to the premenopause group (p = 0.076 and p = 0.025, respectively).

**Table 1:** Basic clinical characteristics and intergroup differences of participants.

Index	Premenopause	Transition phase	Postmenopause	P-value (Pre vs Trans)	P-value (Trans vs post)	P-value (Pre vs post)
Age (Year)	34.150 ± 6.642	52.267 ± 4.201	57.240 ± 3.459	<0.001	<0.001	<0.001
FSH(IU/L)	14.588 ± 5.539	32.213 ± 13.768	50.861 ± 17.719	<0.001	<0.001	<0.001
LH (IU/L)	11.740 ± 4.278	24.695 ± 7.624	41.003 ± 8.756	<0.001	<0.001	<0.001
E2 (pmol/L)	231.304 ± 91.241	172.876 ± 73.685	55.742 ± 15.728	<0.01	<0.001	<0.001
Height (cm)	163.373 ± 4.124	161.163 ± 5.38	163.390 ± 4.883	0.084	0.104	0.989
Weight (kg)	62.253 ± 11.645	69.100 ± 16.766	60.710 ± 9.061	0.076	<0.05	0.575
BMI	23.332 ± 4.321	26.582 ± 6.287	22.719 ± 3.082	<0.05	<0.01	0.537
WBC (10^9)	6.000 ± 1.554	5.507 ± 1.238	8.516 ± 8.614	0.186	0.068	0.127
FBG (mmol/L)	5.312 ± 0.315	5.344 ± 0.292	5.351 ± 0.565	0.690	0.957	0.751
TG (mmol/L)	1.546 ± 1.093	1.476 ± 0.591	1.869 ± 1.347	0.763	0.155	0.320
CHOL (mmol/L)	4.875 ± 0.855	5.825 ± 0.688	5.427 ± 0.989	<0.001	0.081	<0.05
HDLC (mmol/L)	1.717 ± 0.442	1.705 ± 0.262	1.755 ± 0.526	0.903	0.646	0.763
LDLC (mmol/L)	2.763 ± 0.596	3.481 ± 0.681	3.105 ± 0.761	<0.001	0.052	0.062

Data are presented as mean ± standard deviation. Intergroup comparisonswere performed using one-way ANOVA followed by Tukey’s *post hoc* test when applicable, with statistical significance set at p < 0.05. The premenopause group represents premenopausal women, the Transition group represents women in the menopause transition stage, and the Post group represents postmenopausal women. Sample size for each group: N = 30. BMI, body mass index; CHOL, total cholesterol; E2, estradiol; FBG, fasting blood glucose; FSH, follicle-stimulating hormone; HDLC, high-density lipoprotein cholesterol; LDLC, low-density lipoprotein cholesterol; LH, luteinizing hormone; TG, triglycerides; WBC, white blood cell count.

White blood cell count (WBC) and fasting blood glucose (FBG) showed relatively stable patterns (p > 0.05). Total cholesterol (CHOL) and low-density lipoprotein cholesterol (LDLC) levels were significantly higher in both the menopause transition and postmenopause groups compared to premenopause (p < 0.001 and p = 0.027, respectively). Conversely, triglycerides (TG) and high-density lipoprotein cholesterol (HDLC) showed no significant differences across the groups, suggesting limited association of menopausal status on these parameters.

### 3.2 Lipid class characteristics in menopause transition and postmenopause

**Figure 1:**
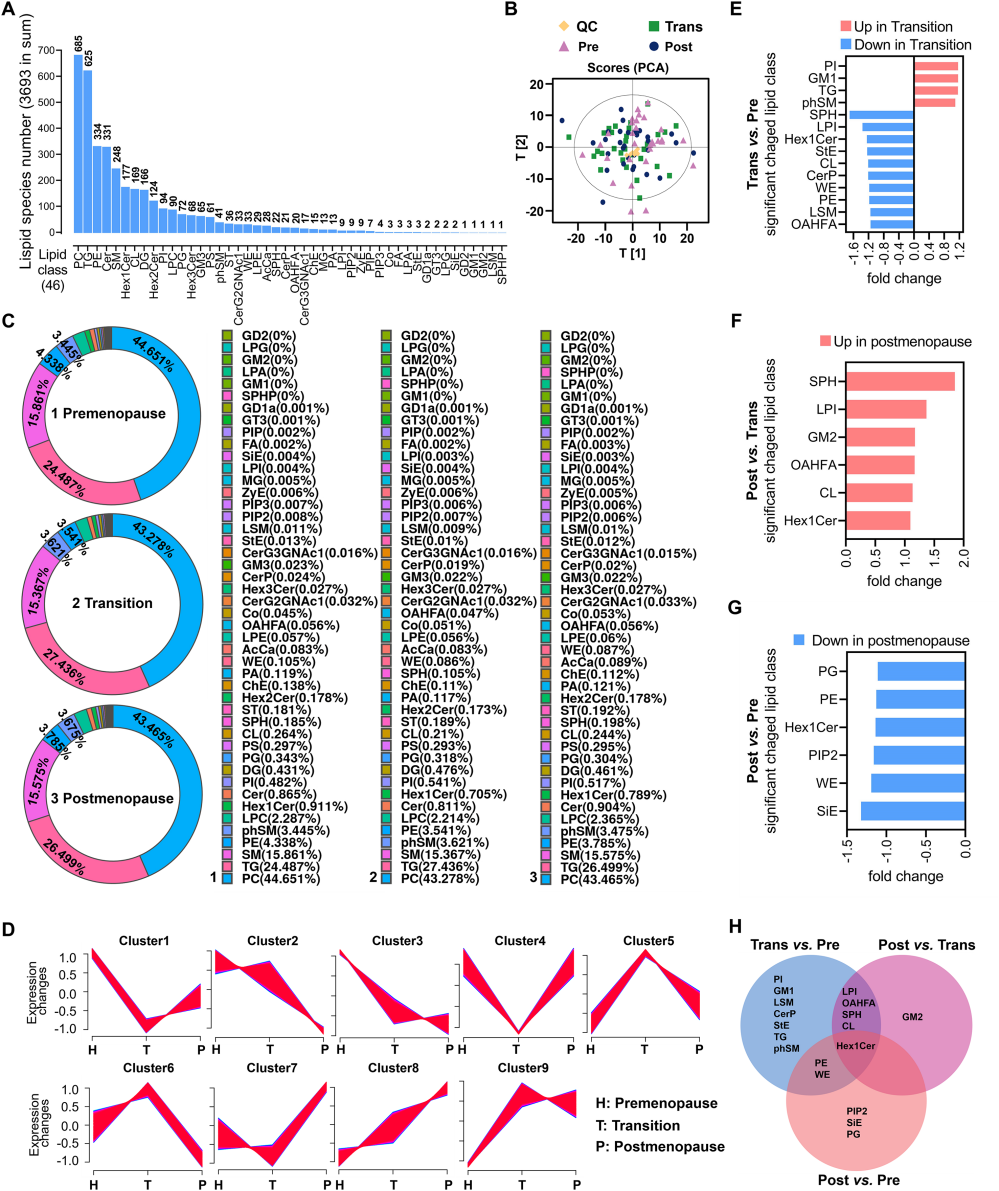
Distribution and characteristics of lipid classes in menopause transition and postmenopause. **(A)** Bar chart showing the distribution of 46 identified lipid classes and their corresponding lipid species, ranked by abundance. **(B)** PCA plot illustrating the distribution of samples (premenopause, menopause transition, and postmenopause) and QC samples in principal component space, reflecting overall differences in lipid metabolism. **(C)** Donut charts depicting the composition of lipid classes in the three groups, showing differences in proportional distribution. **(D)** Trend clustering analysis performed using Mfuzz software with fuzzy c-means algorithm, showing normalized expression patterns of lipid species across groups. Different colors represent distinct clusters. **(E-G)** Bar charts showing significantly different lipid classes between the groups: premenopause vs. menopause transition **(E)**, menopause transition vs. postmenopause **(F)**, and premenopause vs. postmenopause **(G)**. **(H)** Venn diagram illustrating the overlap and specificity of significantly different lipid classes among the three groups.

A total of 46 lipid classes and their corresponding lipid species were identified, with PC (685 species), TG (625 species), PE (334 species), Cer (331 species), and SM (248 species) being the most abundant lipid classes (**Figure 1A**). Principal component analysis (PCA) revealed distinct separation of lipidomic profiles among the three groups, with the distribution of quality control (QC) samples confirming the stability of the data (**Figure 1B**). Donut charts illustrated the compositional characteristics of lipid classes in the three groups, highlighting PC, TG, SM, PE, and phSM as the five most prevalent classes. Notably, differences in the proportions of PE and phSM indicated group-specific variations in lipidomic composition (**Figure 1C**). Trend clustering analysis, based on lipid expression patterns, categorized all lipid species into nine distinct modules. These modules captured dynamic changes in lipid levels across groups. Some clusters showed significant upregulation during the menopause transition followed by a decline postmenopause, while others exhibited sustained increases or decreases in the postmenopause group (**Figure 1D**). Differential lipid analysis revealed significant changes in lipid classes between groups. Compared with the premenopause group, the menopause transition group exhibited upregulation of PI, GM1, TG, and phSM, and downregulation of SPH, LPI, and Hex1Cer, with a total of 14 lipid classes showing significant differences (**Figure 1E**). Between the menopause transition and postmenopause groups, six lipid classes, including SPH and LPI, were significantly upregulated in the postmenopause group (**Figure 1F**). Comparing the premenopause group and the postmenopause group, six lipid classes, including PG and PE, were significantly downregulated in the postmenopause group (**Figure 1G**). Venn diagram analysis of differential lipid classes across the three groups identified lipid classes with transition-specific changes, postmenopause-specific changes, and those commonly altered in both stages (**Figure 1H**). These patterns suggest stage-specific alterations in lipid metabolism during menopause, with certain lipid classes potentially serving as biomarkers for menopause-associated metabolic changes.

### 3.3 Intergroup comparisons of key lipid classes in menopause transition and postmenopause

**Table 2** and **Table 3** summarizes the mean levels and statistical differences of 18 lipid classes (PI, GM1, LSM, CerP, StE, TG, phSM, LPI, OAHFA, SPH, CL, PE, WE, PIP2, SiE, PG, GM2, and Hex1Cer) among the premenopause, menopause transition, and postmenopause groups. Ten lipid classes—LPI, Hex1Cer, OAHFA, PE, WE, PIP2, CL, PI, GM1, and SiE—showed statistically significant intergroup differences based on one-way ANOVA (**Table 2**). Notably, Hex1Cer levels decreased significantly from the premenopause group to the transition group (p < 0.001), but then rebounded in the postmenopause group to levels significantly higher than both earlier stages (Post vs. Pre: p < 0.05), indicating a biphasic regulatory pattern. Similarly, LPI, OAHFA, CL, and PE demonstrated significant changes during the menopause transition, with some showing reversal or stabilization in the postmenopausal stage. GM1 and SiE showed increases during the transition phase but subsequently declined in the postmenopausal stage, returning to levels comparable to the premenopausal group. For example, GM1 significantly increased from premenopause to transition group (p < 0.05), while SiE showed a decreasing trend from transition to postmenopause group (p < 0.05) (**Table 3**). These findings suggest that the dynamic changes in different lipid classes across the menopause transition and postmenopause stages reflect the complexity and stage-specific characteristics of lipid regulatory mechanisms in the body.

**Table 2:** Summary statistics and ANOVA results for 18 lipid classes across menopausal stages.

Lipid Class	Premenopause	Transition phase	Postmenopause	F-value	ANOVA p-value
PI	40840968.28 ± 7793650.30	47577576.40 ± 9835019.52	44509970.61 ± 10803989.00	3.73	<0.05
GM1	35097.84 ± 8062.96	40877.84 ± 10951.55	36856.32 ± 6620.73	3.45	<0.05
LSM	934175.45 ± 212709.73	812249.93 ± 204281.40	835275.69 ± 246458.55	2.56	0.083
CerP	2043543.87 ± 787410.44	1692141.48 ± 416788.90	1738945.78 ± 587997.99	2.88	0.061
StE	1138649.49 ± 447280.51	920917.54 ± 361576.96	1017153.81 ± 507229.28	1.82	0.167
TG	2073584616.93 ± 605877063.45	2411518987.83 ± 663799851.36	2280960797.90 ± 671487407.85	2.08	0.131
phSM	291768228.99 ± 46480895.25	318294299.10 ± 54394711.31	299143931.16 ± 44539206.43	2.38	0.099
LPI	381044.13 ± 116422.48	280095.70 ± 111788.69	383705.54 ± 86888.07	9.34	<0.001
OAHFA	4751251.00 ± 731775.26	4138148.74 ± 823618.37	4837645.88 ± 687057.29	7.76	<0.001
SPH	15650759.61 ± 11034846.64	9200961.05 ± 7123932.85	17035537.44 ± 18815493.42	2.99	0.055
CL	22341258.78 ± 7515620.14	18485592.16 ± 3075789.37	20967685.50 ± 4667974.66	3.92	<0.05
PE	367360990.42 ± 91583517.27	311221814.27 ± 55241109.65	325828290.81 ± 58175134.71	5.15	<0.01
WE	8893574.18 ± 2547662.37	7518776.83 ± 1938999.90	7463829.60 ± 1854572.35	4.31	<0.05
PIP2	640496.97 ± 129398.79	596235.86 ± 127729.60	552437.94 ± 103045.33	3.99	<0.05
SiE	370733.90 ± 146588.53	333878.07 ± 121852.62	280467.50 ± 140546.30	3.31	<0.05
PG	29032345.08 ± 5833383.18	27913701.11 ± 4543521.49	26171185.27 ± 3099013.46	2.91	0.059
GM2	16391.68 ± 3610.57	15331.38 ± 4854.50	17984.97 ± 3961.45	3.07	0.051
Hex1Cer	77160298.36 ± 17933115.60	61967801.76 ± 9531093.98	67892496.53 ± 12383024.75	9.33	<0.001

Data are presented as mean ± standard deviation (SD) for each lipid class across the premenopause, menopause transition, and postmenopause groups (N = 30 per group). Statistical differences were assessed using one-way analysis of variance (ANOVA). The F-statistic and overall P-value are reported for each lipid class.

**Table 3:** *Post hoc* pairwise comparisons of lipid classes using Tukey’s HSD test.

Lipid	Group1	Group2	Meandiff	p-adj	Lower	Upper	Reject
PI	Post	Pre	-3669002	0.302	-9554957	2216953	FALSE
PI	Post	Trans	3067606	0.432	-2818349	8953561	FALSE
PI	Pre	Trans	6736608	<0.05	850653.1	12622563	TRUE
GM1	Post	Pre	-1758.48	0.716	-7134.96	3618.005	FALSE
GM1	Post	Trans	4021.521	0.181	-1354.96	9398.002	FALSE
GM1	Pre	Trans	5779.997	<0.05	403.5159	11156.48	TRUE
LSM	Post	Pre	98899.76	0.201	-37717.1	235516.6	FALSE
LSM	Post	Trans	-23025.8	0.915	-159643	113591.1	FALSE
LSM	Pre	Trans	-121926	0.090	-258542	14691.32	FALSE
CerP	Post	Pre	304598.1	0.141	-74838.1	684034.3	FALSE
CerP	Post	Trans	-46804.3	0.954	-426241	332631.9	FALSE
CerP	Pre	Trans	-351402	0.075	-730839	28033.81	FALSE
StE	Post	Pre	121495.7	0.540	-151091	394082.8	FALSE
StE	Post	Trans	-96236.3	0.678	-368823	176350.8	FALSE
StE	Pre	Trans	-217732	0.144	-490319	54855.16	FALSE
TG	Post	Pre	-2.1E+08	0.433	-6.1E+08	1.91E+08	FALSE
TG	Post	Trans	1.31E+08	0.716	-2.7E+08	5.29E+08	FALSE
TG	Pre	Trans	3.38E+08	0.113	-6.1E+07	7.37E+08	FALSE
phSM	Post	Pre	-7375702	0.827	-3.7E+07	22581982	FALSE
phSM	Post	Trans	19150368	0.285	-1.1E+07	49108052	FALSE
phSM	Pre	Trans	26526070	0.093	-3431614	56483754	FALSE
LPI	Post	Pre	-2661.4	0.995	-67818.2	62495.41	FALSE
LPI	Post	Trans	-103610	<0.001	-168767	-38453	TRUE
LPI	Pre	Trans	-100948	<0.001	-166105	-35791.6	TRUE
OAHFA	Post	Pre	-86394.9	0.896	-547927	375137.3	FALSE
OAHFA	Post	Trans	-699497	<0.01	-1161029	-237965	TRUE
OAHFA	Pre	Trans	-613102	<0.01	-1074634	-151570	TRUE
SPH	Post	Pre	-1384778	0.914	-9541271	6771715	FALSE
SPH	Post	Trans	-7834576	0.063	-1.6E+07	321916.7	FALSE
SPH	Pre	Trans	-6449799	0.149	-1.5E+07	1706694	FALSE
CL	Post	Pre	1373573	0.589	-1955887	4703033	FALSE
CL	Post	Trans	-2482093	0.183	-5811554	847366.8	FALSE
CL	Pre	Trans	-3855667	<0.05	-7185127	-526206	TRUE
PE	Post	Pre	41532700	0.063	-1744809	84810208	FALSE
PE	Post	Trans	-1.5E+07	0.701	-5.8E+07	28671032	FALSE
PE	Pre	Trans	-5.6E+07	<0.01	-9.9E+07	-1.3E+07	TRUE
WE	Post	Pre	1429745	<0.05	114565.2	2744924	TRUE
WE	Post	Trans	54947.23	0.995	-1260232	1370127	FALSE
WE	Pre	Trans	-1374797	<0.05	-2689977	-59618	TRUE
PIP2	Post	Pre	88059.03	<0.05	13771.67	162346.4	TRUE
PIP2	Post	Trans	43797.93	0.342	-30489.4	118085.3	FALSE
PIP2	Pre	Trans	-44261.1	0.335	-118548	30026.26	FALSE
SiE	Post	Pre	90266.4	<0.05	6082.655	174450.1	TRUE
SiE	Post	Trans	53410.56	0.290	-30773.2	137594.3	FALSE
SiE	Pre	Trans	-36855.8	0.551	-121040	47327.91	FALSE
PG	Post	Pre	2861160	<0.05	11380.67	5710939	TRUE
PG	Post	Trans	1742516	0.316	-1107263	4592295	FALSE
PG	Pre	Trans	-1118644	0.619	-3968423	1731135	FALSE
GM2	Post	Pre	-1593.29	0.306	-4163.8	977.2196	FALSE
GM2	Post	Trans	-2653.59	<0.05	-5224.1	-83.0745	TRUE
GM2	Pre	Trans	-1060.29	0.589	-3630.81	1510.217	FALSE
Hex1Cer	Post	Pre	9267802	<0.05	812867.1	17722737	TRUE
Hex1Cer	Post	Trans	-5924695	0.222	-1.4E+07	2530240	FALSE
Hex1Cer	Pre	Trans	-1.5E+07	<0.001	-2.4E+07	-6737562	TRUE

Pairwise comparisons of lipid class levels between groups (postmenopause vs. premenopause, postmenopause vs. transition, premenopause vs. transition) were conducted using Tukey’s honest significant difference (HSD) test following one-way ANOVA. For each comparison, the mean difference, adjusted P-value (p-adj), and significance status are reported. P < 0.05 was considered statistically significant.

### 3.4 Principal component analysis and species-specific differences in lipid metabolism

**Figure 2:**
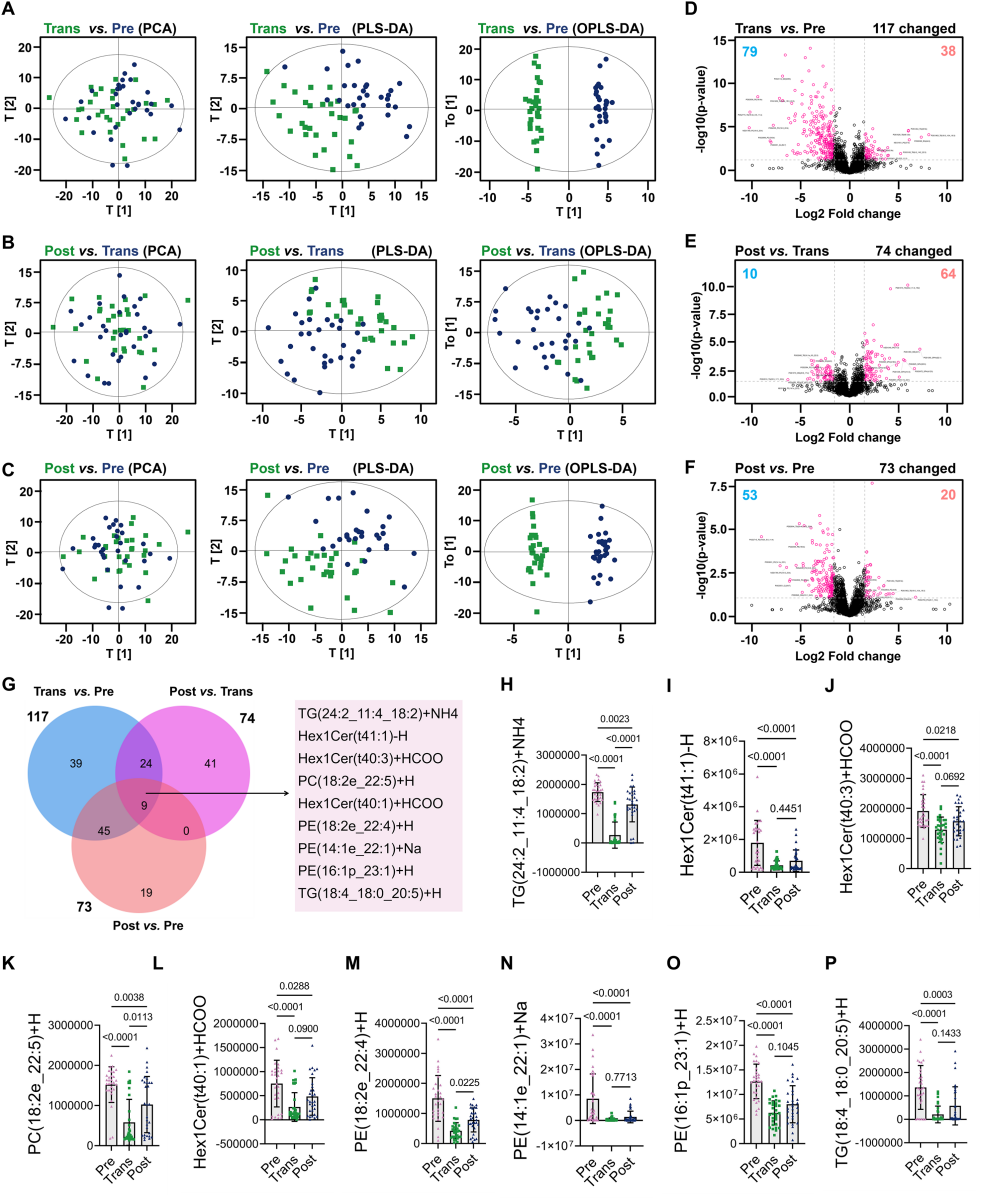
Principal components and species-specific differences in lipid metabolism across menopausal stages. **(A-C)** PCA, PLS-DA, and OPLS-DA plots comparing the premenopause vs. menopause transition groups **(A)**, menopause transition vs. postmenopause groups **(B)**, and premenopause vs. postmenopause groups **(C)**. **(D-F)** Volcano plots showing significantly altered lipid species: 117 species (38 upregulated, 79 downregulated) in premenopause vs. menopause transition **(D)**, 74 species (64 upregulated, 10 downregulated) in menopause transition vs. postmenopause **(E)**, and 73 species (20 upregulated, 53 downregulated) in premenopause vs. postmenopause **(F)**. **(G)** Venn diagram highlighting shared and unique significantly altered lipid species among the three groups, identifying nine common species. **(H-P)** Bar charts showing the mean levels of nine representative lipid species, including TG(24:2_11:4_18:2)+NH4, Hex1Cer(t41:1)-H, and PE(18:2e_22:4)+H, across groups. Statistical differences were assessed using one-way ANOVA and Tukey HSD tests.

Multivariate statistical analyses demonstrated that principal component analysis (PCA) did not show clear separation between the premenopause, menopause transition, and postmenopause groups (**Figures 2A–C**). However, partial least squares discriminant analysis (PLS-DA) and orthogonal PLS-DA (OPLS-DA) provided a clearer differentiation between groups. Notably, OPLS-DA revealed significant separation between the premenopause and menopause transition groups, as well as between the premenopause and postmenopause groups, suggesting pronounced changes in lipidomic profiles during these transitions. In contrast, the separation between the menopause transition and postmenopause groups was less pronounced, indicating that lipidomic changes between these stages were relatively moderate compared to the transition from premenopause to menopause (**Figures 2A–C**). Volcano plot analysis identified the number and trends of significantly altered lipid species between groups (**Figures 2D–F**). Comparing the premenopause and menopause transition groups, 117 lipid species showed significant changes, with 38 species upregulated and 79 downregulated (**Figure 2D**). In the comparison between the menopause transition and postmenopause groups, 74 lipid species exhibited significant changes, with 64 upregulated and 10 downregulated in the postmenopause group (**Figure 2E**). Between the premenopause and postmenopause groups, 73 lipid species were significantly altered, including 20 upregulated and 53 downregulated species (**Figure 2F**). These results indicate that the most extensive lipidomic reorganization occurs during the menopause transition stage (**Figures 2D–F**). Venn diagram analysis highlighted the shared and unique significantly altered lipid species across the three groups, identifying nine common differentially expressed lipid species, such as TG(24:2_11:4_18:2)+NH4 and Hex1Cer(t41:1)-H (**Figure 2G**). While these common species serve as representative markers, unique lipid species may also provide valuable insights as potential biomarkers. Analysis of the nine common lipid species revealed significant differences in lipid metabolism across groups (**Figures 2H–P**). Most of these species, including TG, Hex1Cer, PC, and PE, showed significant downregulation in the menopause transition group compared to the premenopause group. Although some species, particularly those in the TG and PE classes, partially recovered in the postmenopause group, their levels remained below those observed in the premenopause group. At the lipid class level, TG showed an overall increase during both the menopause transition and postmenopause stages, while Hex1Cer and PE levels was lower. In contrast, PC levels did not exhibit significant changes across the stages. These findings highlight discrepancies between trends at the lipid class level and their corresponding species, emphasizing the complexity of lipidomic regulation. Overall, the dynamic changes in lipid species indicate that the menopause transition is a critical stage for lipid metabolic remodeling. Postmenopause appears to be characterized by partial recovery or further adjustment of lipid species, reflecting ongoing metabolic adaptation (**Figures 2H–P**).

### 3.5 Transcriptomic expression characteristics and functional enrichment in leukocytes

**Figure 3:**
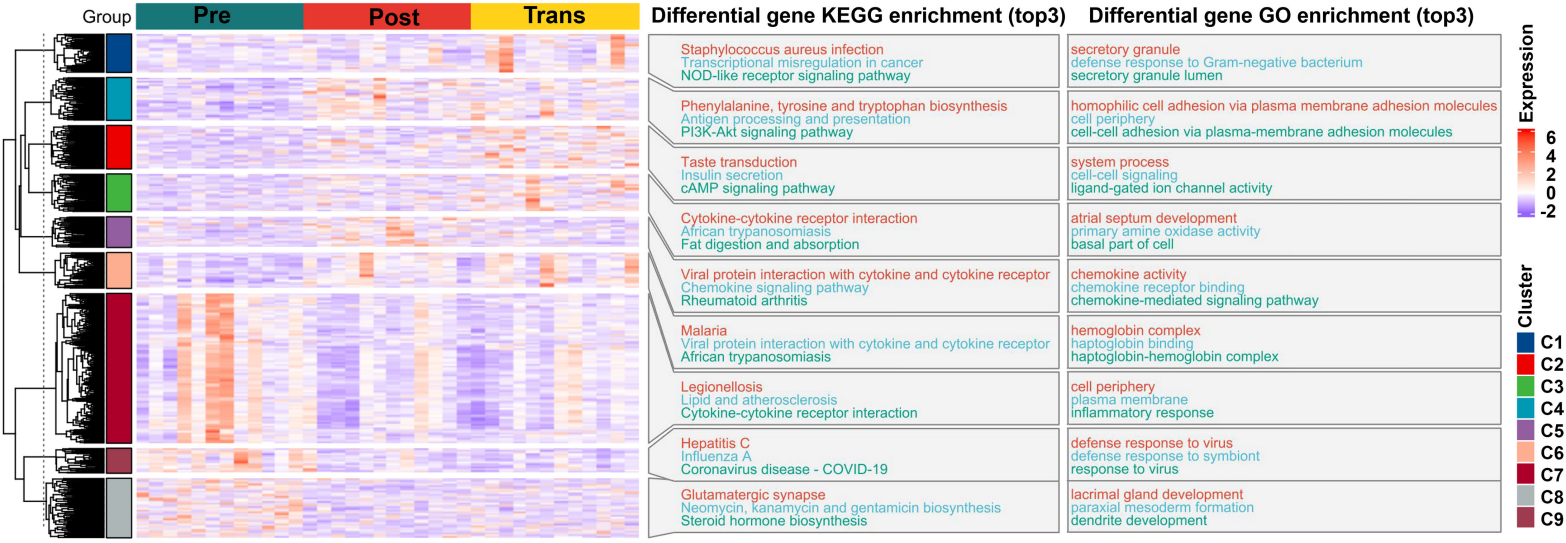
Functional enrichment analysis of transcriptomic clusters reveals key pathways associated with menopausal transition. The heatmap displays the expression profiles of differentially expressed genes (DEGs) grouped into nine clusters based on shared trends across premenopause, menopause transition, and postmenopause stages. For each cluster, the top three significantly enriched Gene Ontology (GO) processes and KEGG pathways are listed.

The transcriptomic data quality was first confirmed by FPKM distribution and uniform read coverage across 36 samples, demonstrating consistent expression levels and sequencing integrity (**Supplementary Figures 1A, B**). A total of 465 DEGs were identified between premenopause and menopause transition, 418 between premenopause and postmenopause, and 148 between menopause transition and postmenopause (**Supplementary Figure 1C**). Venn diagram analysis revealed stage-specific expression patterns, with minimal overlap across comparisons (**Supplementary Figure 1D**). Volcano plots highlighted top up- and downregulated genes in each pairwise comparison (**Supplementary Figures 1E–G**). Clustering analysis grouped DEGs into nine distinct expression modules, with clusters 1–6 showing progressive upregulation and clusters 7–9 showing downregulation (**Supplementary Figure 1H**). The functional characteristics of these nine modules were further summarized by cluster-based KEGG and GO enrichment, as shown in **Figure 3**. KEGG and GO databasesshowed that genes in cluster 7 were significantly enriched in pathways such as Legionellosis, Lipid and atherosclerosis, and Cytokine-cytokine receptor interaction (KEGG), as well as biological processes including cell periphery, plasma membrane, and inflammatory response (GO) (**Figure 3**). These findings underscore the role of these genes in immune and lipid metabolism processes during menopausal stages.

### 3.6 Pathway enrichment and core network analysis of differentially expressed genes

GO enrichment analysis of differentially expressed genes (DEGs) between the premenopause (Pre) and menopause transition (Trans) groups highlighted the top 10 enriched biological processes (BPs), including response to external stimulus, defense response, and immune system process. Additionally, cellular component (CC) enrichment revealed associations with cell periphery and plasma membrane (**Supplementary Figure 2A**). In contrast, DEGs between the menopause transition (Trans) and postmenopause (Post) groups were enriched for molecular functions (MFs) such as chemokine activity and extracellular matrix structural constituent, emphasizing immune regulation and inflammation-related functions (**Supplementary Figure 2B**). The comparison between the premenopause (Pre) and postmenopause (Post) groups showed significant enrichment in BPs such as response to external stimulus, immune response, and defense response, as well as CCs like cell periphery and plasma membrane (**Supplementary Figure 2C**). KEGG enrichment analysis of DEGs identified pathways associated with lipid and atherosclerosis, NF-kappa B signaling pathway, and PPAR signaling pathway, reflecting metabolic and inflammatory regulation across all comparisons (**Supplementary Figures 2D–F**).

**Figure 4:**
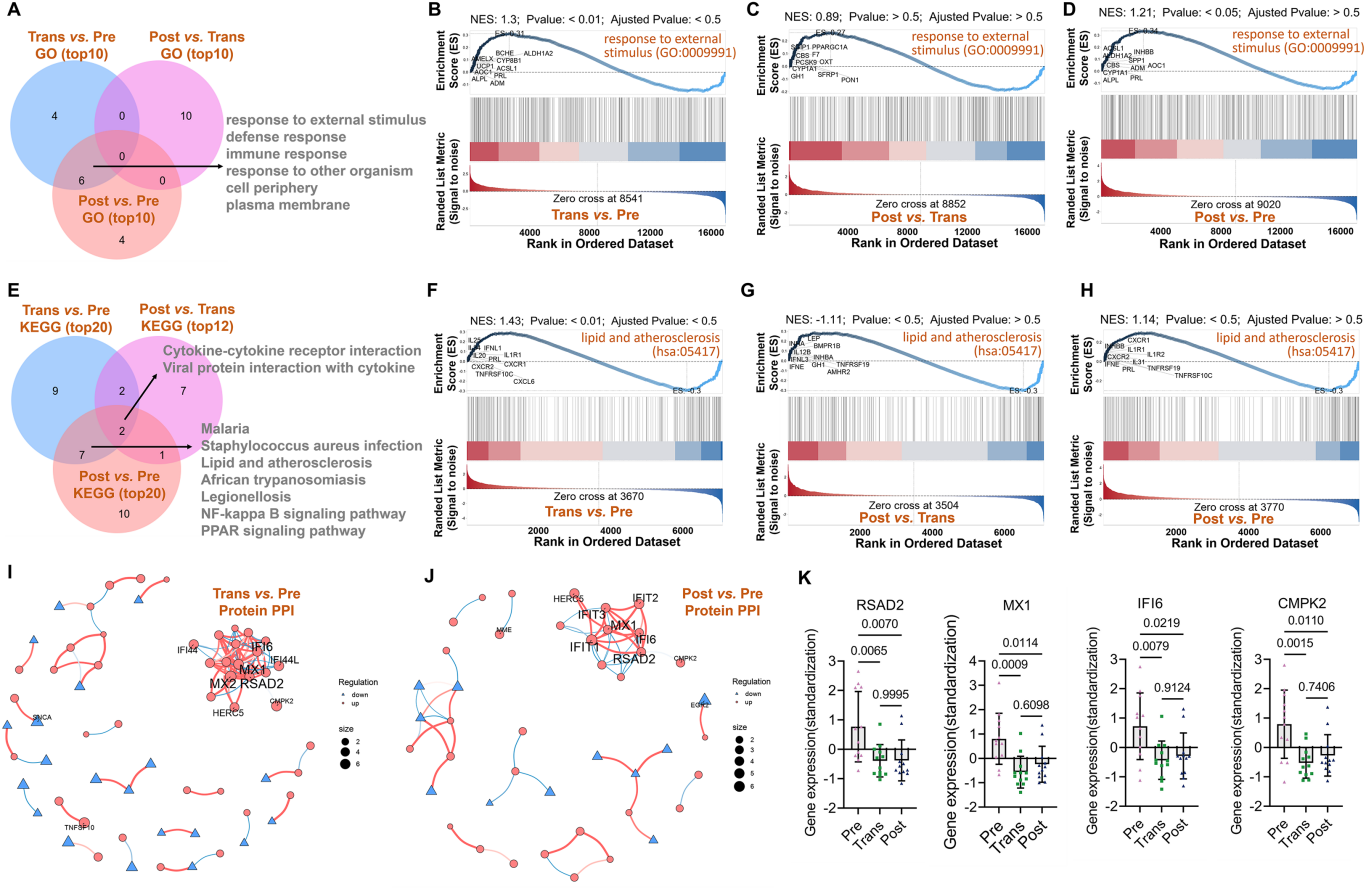
Shared pathway signatures and hub gene network reveal immunometabolic transitions in menopause. **(A)** Venn diagram illustrating shared and specific GO-enriched pathways across the three comparisons. **(B-D)** GSEA analysis of the GO term response to external stimulus for premenopause vs. menopause transition **(B)**, menopause transition vs. postmenopause **(C)**, and premenopause vs. postmenopause **(D)**. **(E)** Venn diagram of shared and specific KEGG-enriched pathways across comparisons. **(F-H)** GSEA analysis of the KEGG pathway Lipid and atherosclerosis for premenopause vs. menopause transition **(F)**, menopause transition vs. postmenopause **(G)**, and premenopause vs. postmenopause **(H)**. **(I, J)** PPI networks of DEGs constructed for premenopause vs. menopause transition **(I)** and premenopause vs. postmenopause **(J)**, with core genes highlighted. **(K)** Bar chart showing expression levels of shared core genes (RSAD2, MX1, IFI6, CMPK2) across groups.

Intersection analysis (**Figure 4A**) revealed six shared enriched signals, including response to external stimulus and defense response, between the premenopause and menopause transition groups, as well as between the premenopause and postmenopause groups. In contrast, the enriched signals between the menopause transition and postmenopause groups were entirely distinct, suggesting specific pathway alterations at different stages. Gene set enrichment analysis (GSEA) of the response to external stimulus pathway (**Figures 4B–D**) showed consistent trends across the three comparisons, with the strongest significance observed in the menopause transition versus premenopause comparison (NES = 1.3, p < 0.01). However, the comparisons between postmenopause and the other groups, while trending similarly, did not reach statistical significance (p > 0.5).

Intersection analysis (**Figure 4E**) highlighted two shared pathways, Viral protein interaction with cytokine and cytokine receptor and Cytokine-cytokine receptor interaction, across the three comparisons. Additionally, seven shared enriched pathways between the premenopause and menopause transition groups and the premenopause and postmenopause groups further supported inflammation and metabolism as key functional alterations during these stages. GSEA of the lipid and atherosclerosis pathway (**Figures 4F–H**) in KEGG showed consistent trends across the GSEA of the response to external stimulus pathway in GO. Protein-protein interaction (PPI) network analysis of DEGs revealed key hub genes in each comparison. For the menopause transition versus premenopause group (**Figure 4I**), core genes identified included RSAD2, MX1, MX2, IFI6, IFI44L, IFI44, HERC5, and CMPK2. For the postmenopause versus premenopause group (**Figure 4J**), the core genes included RSAD2, MX1, IFIT1, IFI6, IFIT2, HERC5, and CMPK2. Shared key genes (RSAD2, MX1, IFI6, and CMPK2) exhibited significantly was lower expression in the menopause transition and postmenopause groups compared to the premenopause group (**Figure 4K**), suggesting their potential as marker genes for the menopause transition phase.

### 3.7 Correlation analysis of key clinical indicators, lipid molecules, and marker genes

**Figure 5:**
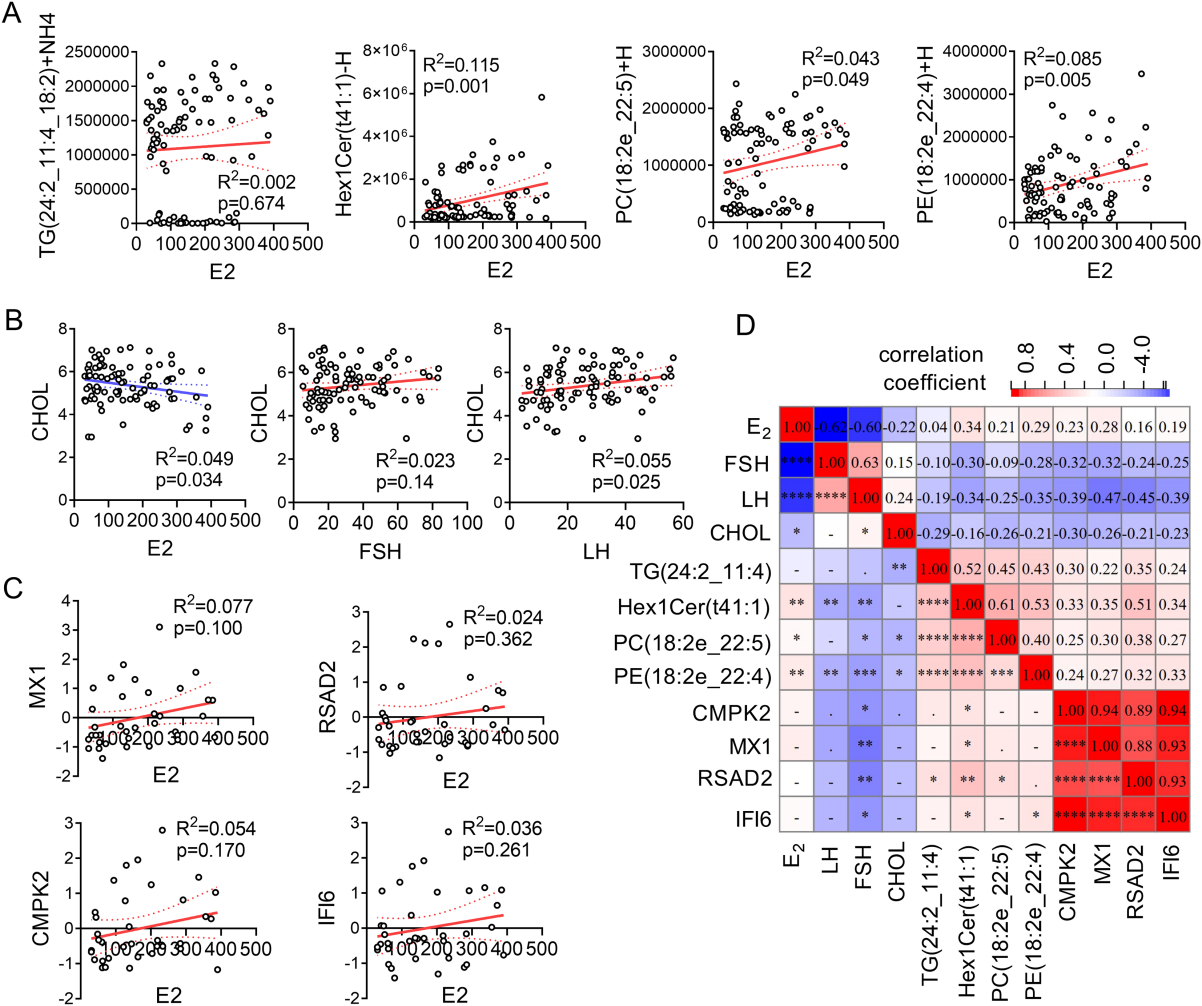
Correlation analysis of clinical indicators, lipid molecules, and transcriptomic signals. **(A)** Correlation analysis between E2 and lipid molecules (TG(24:2_11:4_18:2)+NH4, Hex1Cer(t41:1)-H, PC(18:2e_22:5)+H, PE(18:2e_22:4)+H). **(B)** Correlation analysis between CHOL and clinical indicators (E2, FSH, LH). **(C)** Correlation analysis between E2 and marker genes (CMPK2, MX1, RSAD2, IFI6). **(D)** Heatmap showing pairwise correlations among 12 factors (clinical indicators, lipid molecules, and marker genes). Positive correlations are marked in red, and negative correlations in blue.

Based on the shared differentially expressed lipid species and the PPI-defined hub genes identified in the upstream analyses, we selected representative lipids and genes for focused integrative correlation with key endocrine and metabolic indicators. Estrogen (E2) exhibited significant positive correlations with three lipid molecules—Hex1Cer(t41:1)-H, PC(18:2e_22:5)+H, and PE(18:2e_22:4)+H. However, no significant correlation was observed between E2 and TG(24:2_11:4_18:2)+NH4 (**Figure 5A**). Cholesterol (CHOL) was significantly negatively correlated with E2 and positively correlated with luteinizing hormone (LH), but showed no significant correlation with follicle-stimulating hormone (FSH) (**Figure 5B**). E2 demonstrated a positive trend of correlation with the four marker genes—CMPK2, MX1, RSAD2, and IFI6—suggesting potential regulatory relationships. However, due to the relatively small sample size for transcriptomic data (N = 36), these correlations did not reach statistical significance (p > 0.05) (**Figure 5C**). To further explore interactions among key factors, a heatmap was generated to display the pairwise correlation coefficients and significance levels for 12 factors, including 4 clinical indicators (E2, FSH, LH, CHOL), 4 lipid molecules (TG(24:2_11:4_18:2)+NH4, Hex1Cer(t41:1)-H, PC(18:2e_22:5)+H, PE(18:2e_22:4)+H), and 4 marker genes (CMPK2, MX1, RSAD2, IFI6) (**Figure 5D**). Positive correlations are indicated in red, while negative correlations are shown in blue.

## 4 Discussion

This pilot study utilized integrated multi-omics analysis to explore the metabolic and transcriptomic characteristics of women across normal, menopause transition, and postmenopause stages. Unlike previous studies that grouped the perimenopausal continuum into broad categories (15), we analyzed these stages separately to provide a more detailed view of potential biological shifts. Our findings highlight stage-associated molecular differences that may help shape hypotheses for future research but should be interpreted cautiously due to the study’s exploratory nature and limited cohort size.

### 4.1 Lipidomics and characteristics of menopause transition and postmenopause

Lipidomics analysis highlighted significant differences in lipid metabolism during the menopause transition and postmenopause stages, particularly in triglycerides (TG), phospholipids (PC, PE), and sphingolipids (SM, Hex1Cer). Abnormal lipid metabolism was evident during the menopause transition, with these changes intensifying postmenopause. Notable lipid alterations, such as higher TG and lower Hex1Cer during the menopause transition, suggest early metabolic remodeling driven by declining hormone levels (16). Trend clustering analysis further demonstrated stage-specific regulation, with certain lipids significantly upregulated during the transition phase and stabilizing or declining in postmenopause. Most previous studies combined the menopause transition and postmenopause for analysis, potentially overlooking unique metabolic features of the transition phase (17). By emphasizing the menopause transition as a critical window for metabolic abnormalities, this study provides a basis for early intervention strategies.

### 4.2 Transcriptomics and gene expression changes across menopausal stages

Transcriptomic analysis revealed significant gene expression changes associated with inflammation, immune regulation, and lipid metabolism during menopausal stages. While many inflammation-related genes were upregulated postmenopause, consistent with previous findings of increased inflammation in this stage (18), we observed that lipid metabolism-related genes were already altered during the menopause transition. These changes are likely associated with hormonal decline, setting the stage for more severe metabolic dysregulation postmenopause (19). Protein-protein interaction (PPI) network analysis identified key gene modules, with RSAD2, MX1, and IFI6 emerging as critical regulatory factors during the menopause transition and postmenopause. These genes may serve as key modulators of metabolic remodeling, highlighting the importance of identifying early signals specific to the menopause transition (20). By distinguishing between transition and postmenopause stages, this study sheds light on the progressive regulatory associations of hormone fluctuations on gene expression, offering new perspectives for understanding physiological and pathological changes across these stages.

### 4.3 Integration of clinical indicators with multi-omics features

Integrative analysis of clinical indicators, lipid metabolites, and transcriptomic signals revealed potential mechanisms underlying biological changes in menopause transition and postmenopause. Estrogen (E2) was significantly correlated with several lipid molecules (e.g., PC, PE, Hex1Cer) and marker genes (e.g., CMPK2, RSAD2, IFI6), suggesting systemic relationships of hormone fluctuations on metabolism and gene regulation (21). Heatmap analysis further demonstrated positive correlations between E2 and lipid molecules or genes, while cholesterol (CHOL) showed negative correlations with E2, indicating that hormonal decline may mediate core mechanisms of lipid metabolism and gene regulation.

These findings align with previous studies linking hormonal decline in postmenopause to higher risks of cardiovascular and metabolic diseases through enhanced lipid metabolism and inflammation (22, 23). Moreover, this study underscores the substantial impact of the menopause transition on lipid metabolism and gene expression. Both clustering and differential analysis revealed that changes during the transition phase were more pronounced than those in postmenopause, marking the transition as a critical period of metabolic and regulatory remodeling (24). Correlation analysis further highlighted the interconnections between clinical indicators, metabolism, and gene regulation, emphasizing the importance of the menopause transition as a window for intervention.

### 4.4 Innovation and significance

This study’s key innovation lies in its detailed distinction between menopause transition and postmenopause stages, rather than treating the perimenopausal period as a whole. Through combined lipidomics and transcriptomics analyses, this study elucidates the progressive physiological transitions from perimenopause to postmenopause, particularly the stage-specific changes in metabolism and gene expression. Compared to previous studies, our integrated multi-omics approach identifies metabolic and gene expression features potentially associated with menopause-related diseases such as cardiovascular diseases, osteoporosis, and cancer (25–29). These findings not only provide potential biomarkers for early diagnosis during the menopause transition but also lay the foundation for personalized interventions targeting this critical stage.

### 4.5 Limitations and future directions

Despite its strengths, this study has several limitations that should be considered when interpreting the findings. First, the relatively modest sample size may constrain the robustness and external generalizability of specific metabolic and gene expression features. Second, the analysis focused on circulating lipid metabolites and leukocyte-derived transcripts, which represent systemic readouts but may not fully capture tissue-specific metabolic remodeling in endocrine-responsive organs such as adipose tissue and skeletal muscle.

More importantly, this study is inherently limited by its cross-sectional design. Because menopausal stage is biologically coupled with chronological aging, the molecular differences observed here should be interpreted primarily as stage-associated phenotypes rather than definitive hormone-driven causal effects. Although we grouped participants using the STRAW + 10 staging criteria, which emphasize hormonal and menstrual parameters, age-related metabolic and inflammatory shifts remain intrinsically intertwined with hormonal transition and cannot be completely disentangled in the present framework. In this context, some of the observed lipid and gene expression alterations may reflect age-dependent baseline remodeling rather than menopause-specific mechanisms.

In addition, the integrative depth of the current multi-omics framework is constrained by both cohort size and platform heterogeneity. Although transcriptomic data may be more amenable to cross-cohort harmonization, lipidomic and metabolomic profiles are highly sensitive to analytical platforms, batch effects, and pre-analytical conditions, which substantially limits direct data-level integration across studies. Moreover, most existing longitudinal menopause cohorts are symptom-oriented and do not provide molecular omics stratified by STRAW + 10 biological staging, making external molecular validation challenging. Therefore, the present analysis should be viewed as an internally consistent, stage-specific molecular characterization rather than a fully generalizable omics atlas.

In addition, many historical longitudinal menopause cohorts were established under substantially different nutritional, lifestyle, and cardiometabolic backgrounds than those of contemporary populations. Ongoing population-level transitions in obesity prevalence, physical activity patterns, and metabolic health may therefore reshape the molecular and clinical phenotype of menopause, and should be considered when interpreting cross-generational comparisons.

Finally, the absence of longitudinal follow-up precludes direct assessment of the temporal dynamics and causal trajectories of metabolic and gene regulatory changes across the menopausal transition. Future studies should prioritize larger, longitudinally followed cohorts, integrate multi-omics profiling across multiple metabolically relevant tissues, and incorporate age-adjusted or age-matched analytical strategies. Incorporation of metabolic flux–oriented approaches (e.g., tracer-based studies) will further improve mechanistic resolution. Such designs will be essential to disentangle endocrine-driven from age-driven mechanisms and to refine clinically meaningful biomarkers for early intervention in menopause-related metabolic risk.

## 5 Conclusion

This exploratory analysis revealed stage-associated differences in lipid metabolism and gene expression across premenopausal, transition, and postmenopausal women. The menopause transition stage demonstrated the most marked alterations, particularly in inflammation- and lipid-related pathways. These observations suggest coordinated shifts in hormone levels, lipid molecules, and transcriptomic patterns. However, due to the small sample size and cross-sectional design, these findings should be considered hypothesis-generating. Larger, longitudinal studies are necessary to validate these associations and distinguish menopause-related effects from age-related variation.

## Data Availability

Data supporting the findings of this study are available from the corresponding author upon reasonable request.
