# Supplementary material for: Preliminary lipidomics and transcriptomics reveals stage-specific dynamic metabolic patterns from menopause transition to postmenopause
Source: Front Endocrinol (Lausanne). 2026 Mar 3;17:1726161. doi: 10.3389/fendo.2026.1726161 (PMC12991988; doi:10.3389/fendo.2026.1726161)
Supplement: Supplementary file 1 [file DataSheet1.docx]

**
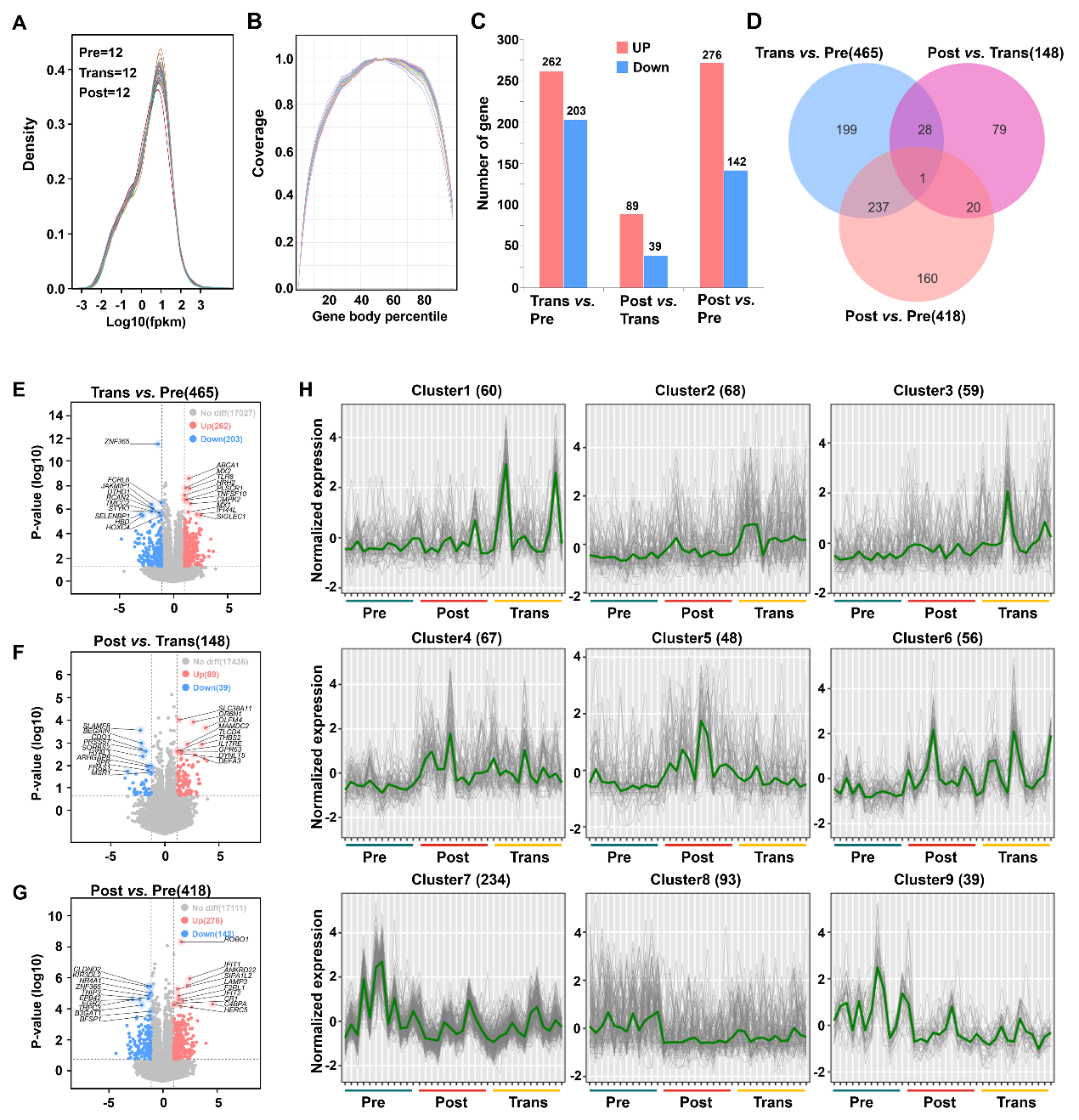
**

**Figure S1. Transcriptomic data quality, DEG summary, and clustering characteristics across menopausal stages.** (A) FPKM density distribution showing gene expression levels across 36 samples in premenopause, menopause transition, and postmenopause groups, reflecting overall expression patterns. (B) Gene coverage uniformity analysis showing 5' to 3' sequence coverage in all samples, confirming uniformity. (C) Bar chart summarizing the number of differentially expressed genes (DEGs) identified in pairwise comparisons: premenopause vs. menopause transition, menopause transition vs. postmenopause, and premenopause vs. postmenopause. (D) Venn diagram of DEGs showing overlap and group-specific differences among comparisons. (E-G) Volcano plots highlighting significantly upregulated and downregulated genes in each comparison, with top 10 genes annotated. (H) Trend analysis of DEGs grouped into nine clusters based on expression patterns across the three groups.


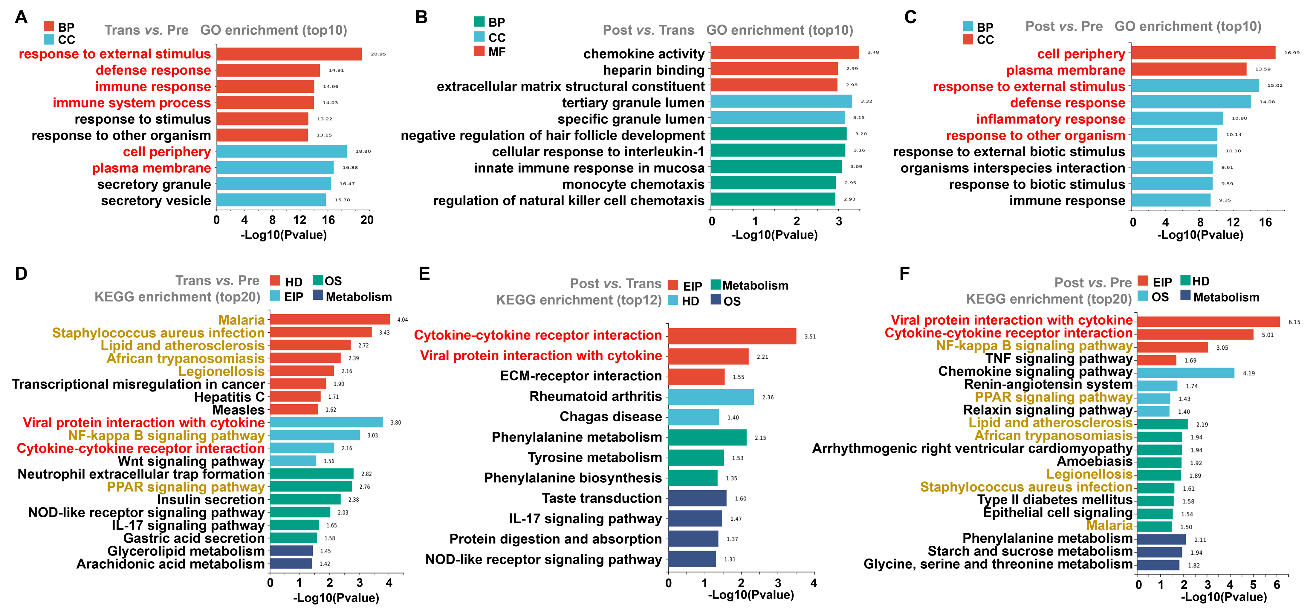


**Figure S2. GO and KEGG enrichment of differentially expressed genes across menopausal stages.** (A-C) Top 10 GO-enriched pathways for DEGs in comparisons of premenopause vs. menopause transition (A), menopause transition vs. postmenopause (B), and premenopause vs. postmenopause (C). (D-F) Top 20 KEGG-enriched pathways for DEGs in comparisons of premenopause vs. menopause transition (D), menopause transition vs. postmenopause (E), and premenopause vs. postmenopause (F).
